# Genetic and environmental contributions to variation in plasma phosphorylated tau 217

**DOI:** 10.1002/alz.70906

**Published:** 2025-11-13

**Authors:** Rebecca Z. Rousset, Conor V. Dolan, David H. Wilson, Lisanne in ‘t Veld, Lannie Ligthart, Charlotte E. Teunissen, Eco J. C. de Geus, Anouk den Braber

**Affiliations:** ^1^ Neurochemistry Laboratory, Department of Clinical Chemistry, Amsterdam UMC Vrije Universiteit Amsterdam, Amsterdam Neuroscience Amsterdam the Netherlands; ^2^ Department of Biological Psychology, Faculty of Behavioural and Movement Sciences Vrije Universiteit Amsterdam Amsterdam the Netherlands; ^3^ Quanterix Corp Billerica Massachusetts USA; ^4^ Alzheimer Center, Department of Neurology, Amsterdam UMC Vrije Universiteit Amsterdam, Amsterdam Neuroscience Amsterdam the Netherlands

**Keywords:** biometric twin model, cognitively healthy population, environmental factors, genetic factors, phosphorylated tau 217, plasma biomarkers, sex differences

## Abstract

**INTRODUCTION:**

Plasma phosphorylated tau 217 (p‐au217) is a promising Alzheimer's disease (AD) biomarker. Little is known about the causes of variance in p‐tau217 concentrations in cognitively unimpaired populations.

**METHODS:**

The present sample included cognitively healthy twins and their family members (*n* = 6495). Biometric twin models were used to determine relative genetic and environmental contributions to variance in p‐tau217, and genetic and environmental correlations between p‐tau217 and neurofilament light chain (NfL), glial fibrillary acidic protein (GFAP), and amyloid beta 42/40 (Aβ42/40).

**RESULTS:**

Genetic contributions accounted for 42% (males) and 41% (females) of variance in p‐tau217. Half of the genetic effects were sex specific. Genetic and environmental contributions to p‐tau217 were partially shared with NfL, GFAP, and Aβ42/40, but different patterns were found in males and females.

**DISCUSSION:**

P‐tau217 concentrations are moderately heritable. Sex‐specific genetic influences are found to act on p‐tau217. In cognitively unimpaired participants, p‐tau217 and Aβ42/40 reflect different biological processes.

**Highlights:**

- Phosphorylated tau (p‐tau)217 concentrations are substantially heritable (> 41%).
- Genetic contributions to variation in plasma p‐tau217 were found to be sex specific.
- Genetic and environmental correlations were found between p‐tau217 and Alzheimer's disease biomarkers.
- P‐tau217 reflects different processes in cognitively unimpaired males and females.

## 1 BACKGROUND

Alzheimer's disease (AD) represents an escalating global health crisis, and is the leading cause of dementia and a major contributor to mortality worldwide.^1^, ^2^ Early diagnosis of AD can be challenging due to its insidious onset and the overlap of initial symptoms with normal aging or other conditions.^3^, ^4^ However, timely diagnosis is increasingly critical as emerging novel treatments appear to be most effective when initiated in the early disease stages, before substantial and irreversible neuronal damage has occurred.^5^

Plasma biomarkers are a non‐invasive and cost‐effective tool to detect AD pathology.^4^, ^6^ One such biomarker is phosphorylated tau (p‐tau), specifically the isoforms 231 (p‐tau231), 181 (p‐tau181), and 217 (p‐tau217), with p‐tau217 having the best diagnostic performance of the three.^6^, ^7^, ^8^, ^9^, ^10^, ^11^, ^12^, ^13^, ^14^ Plasma p‐tau217 has demonstrated great potential to discriminate between individuals with and without amyloid pathology (amyloid beta [Aβ]+ and Aβ–, respectively), even in the pre‐symptomatic stages of AD.^15^, ^16^ Considering its current and future potential clinical uses, a deeper understanding of factors contributing to individual variability in plasma p‐tau217 concentrations is paramount.

One key approach to achieving this understanding is to determine the relative contributions of genetic and environmental factors to the variance in plasma p‐tau217 levels. Indeed, a pre‐print study by Saari et al.^17^ in a twin cohort (*n* = 147 twin pairs) estimated the genetic contribution to plasma p‐tau217 concentration to be 56%. However, the limited sample size precluded the investigation of potential sex‐specific effects.

Previous studies have reported that the association of p‐tau217 and p‐tau181 with clinical outcomes, such as cognitive decline and brain atrophy, was stronger in females than in males.^18^, ^19^ This may be a reflection of AD sex differences, for example, in AD incidence, with two thirds of people with AD being female.^20^ Interestingly, a number of X‐linked genes have been connected to female‐specific cognitive decline and tau accumulation,^21^ which could be reflected in plasma p‐tau217 concentrations. Alternatively, it could be the result of sex differences relating to p‐tau217 concentrations independent of AD pathology. However, as p‐tau217 is a relatively new biomarker, not much is known about sex differences in p‐tau217 concentrations outside of clinical AD.

Beyond examining individual contributions, investigating the correlations between genetic and environmental factors influencing multiple biomarkers can provide insights into shared biological pathways. Significant correlations in both genetic and environmental influences between two biomarkers suggest they may be regulated by common underlying mechanisms. In our study cohort, we previously investigated the genetic and environmental contributions to plasma levels of neurofilament light chain (NfL), a non‐specific marker of neurodegeneration;^22^, ^23^, ^24^, ^25^ glial fibrillary acidic protein (GFAP), a marker of astrocyte activation and neuroinflammation;^23^, ^25^, ^26^, ^27^, ^28^, ^29^ and the Aβ42/40 ratio, a marker for amyloid accumulation in the brain.^30^, ^31^

Relative genetic contributions to NfL, GFAP, and Aβ42/40 were estimated at 42%, 60%, and 16%, respectively, along with a genetic correlation of 0.32 and an environmental correlation of 0.40 between NfL and GFAP. Notably, no significant correlations with Aβ42/40 or sex differences in these parameters were detected.^32^ Here, we extend these findings by estimating the correlations between the genetic and environmental factors contributing to variance in p‐tau217, and those influencing plasma NfL, GFAP, and Aβ42/40 concentrations.

The primary aim of the present study is to estimate the relative contributions of genetic and environmental factors to the variance in plasma p‐tau217 within a cognitively unimpaired cohort. Recognizing the established sex differences in AD incidence, with females disproportionately affected, and the sex differences in the association between p‐tau217 and AD‐related clinical outcomes, we also investigate potential sex‐specific variations in these contributing factors. Furthermore, we seek to elucidate the extent to which the genetic and environmental influences on p‐tau217 are shared with those affecting other key plasma AD biomarkers—NfL, GFAP, and the Aβ42/40 ratio—to gain insights into the source of the relationship between these biomarkers, using family twin‐based biometric models.^33^, ^34^, ^35^, ^36^, ^37^

## 2 METHODS

### 2.1 Cohort

**RESEARCH IN CONTEXT**

1. **Systematic review**: Plasma phosphorylated tau (p‐tau) 217 concentrations are elevated in individuals with Alzheimer's disease pathology. However, it is not well understood what contributes to variation in p‐tau217 concentrations in cognitively healthy individuals.
2. **Interpretation**: Genetic contributions to variation in plasma p‐tau217 are substantial (> 41%), and are in part due to sex‐specific genetic influences. Additionally, p‐tau217 correlated moderately with neurofilament light chain and glial fibrillary acidic protein. Correlations with the amyloid beta (Aβ)42/40 ratio were weak.
3. **Future directions**: Sex differences in genetic contributions to p‐tau217 concentrations are significant in cognitively unimpaired individuals. A low correlation between p‐tau217 and Aβ42/40 indicates these biomarkers are part of different biological processes in the absence of Alzheimer's disease (AD) pathology. Future research should aim to further characterize the sex‐specific genetic contributions to p‐tau217 concentrations, which may relate to the sex differences in the processes leading to AD development.

Data were extracted from the Netherlands Twin Register (NTR), a longitudinal biobank that collects extensive data, including genetic, lifestyle, and health‐related information, from Dutch twins and their family members.^38^ Plasma samples were collected for ≈ 10,000 NTR participants between 2004 and 2011,^39^ with participants spanning an age range of 18 to 98 years old. As the assays used for biomarker measurement have only been validated in older participants, we used an age cutoff of 40 years for the majority of our participants. An exception was made for twins only, who were included from age ≥ 30, as we wanted to enrich our cohort with more twin pairs. These age cutoffs were also chosen to ensure a sufficient age range for studying biomarker variability while minimizing the inclusion of individuals likely to have manifest AD pathology at the time of blood collection, given the typical onset after age 65,^2^ and an AD incidence estimate of 9.8 per 100,000 persons‐years for people between 30 and 64 years old.^40^ Participants were included if they self‐reported being cognitively unimpaired. The included families consisted of twins, their parents, and a maximum of two non‐twin siblings. For families with more than two non‐twin siblings, the siblings closest in age to the twins were included. If no data were available for the twins, the two oldest siblings were chosen. This amounted to the inclusion of 6495 participants from 3307 families.

### 2.2 Biomarker measurement

Plasma samples were collected in the morning after overnight fasting. The procedure has been described in detail elsewhere.^39^ After collection, samples were stored at −20°C prior to measurement. The biomarkers were measured on the Simoa HD‐X using the Janssen p217+ tau assay (Quanterix) for p‐tau217, and the Neurology 4‐Plex E advantage kit (Quanterix) for NfL, GFAP, Aβ42, and Aβ40. Each Simoa HD‐X run consists of about three plates, or ≈ 250 samples. Samples belonging to the same family were randomized across all runs to ensure that family resemblances would not be inflated due to run‐specific influences. For p‐tau217, the first 337 samples were measured in duplicate as per the kit instructions. The remaining 6158 samples were measured in single measurements based on quality measures from the duplicate runs (mean sample difference between first and second measurement: 0.0024 pg/mL, 95% confidence interval [CI]: –0.0027 to 0.0076, Figure S1 in supporting information). Samples were measured over 29 runs, with a quality control inter‐assay coefficient of variability of 9.1%, which is within the accepted range (< 20%).^41^ NfL, GFAP, Aβ42, and Aβ40 were measured across 33 runs with good inter‐assay coefficients of variability for the quality controls of all four markers (NfL: 4.5%, GFAP: 4.6%, Aβ42: 5.2%, and Aβ40: 4.1%).^32^ To account for samples being stored at −20°C for an extended period of time, which can lead to sample degradation,^42^ we compared our biomarker concentrations to those obtained in a separate cohort for which samples were stored at −80°C and subsequently measured using the same kit lot number and on the same Simoa HD‐X as the samples of our own cohort. We observed an average decrease of 20% for p‐tau217 concentrations in our samples, as well as an increase of 11% for Aβ42/40 concentrations, and an increase of 26% for both NfL and GFAP. Regression to the median was used to derive correction factors to approximate biomarker concentrations before degradation, and these correction factors were applied to the biomarker concentrations before further analysis.

### 2.3 Statistical analysis

Statistical analyses were performed using the OpenMx package in R 4.3.2. A log‐transformation was applied to the NfL, GFAP, and p‐tau217 measurements to render the data approximately normal. All biomarker distributions were standardized using *z* score transformation to put them on a common scale for variance component estimation. Outliers were identified in two steps. First, boxplots of each biomarker were made to visually inspect the distribution. Here, it was determined that there were outlier concentrations for NfL, GFAP, Aβ42, and Aβ40, but not p‐tau217. As a second step, outliers for these biomarkers were then defined as deviating –/+ 3 standard deviations from the mean for NfL (*n* = 15, 0.2%), GFAP (*n* = 17, 0.3%), Aβ42 (*n* = 10, 0.2%), and Aβ40 (*n* = 16, 0.3%), and were excluded from the analyses. Data transformation and outlier identification were performed before splitting the data by sex and zygosity of the twins. Biomarker levels were corrected for possible age and sex influences by adding age, sex, and age x sex as covariates to the twin and family models in OpenMx. Specific hypotheses concerning the parameters in the models were tested by comparing nested models using the likelihood ratio test, with a *p* value < 0.05 considered indicative of a significantly worse fit (implying that the parameter constraint informed by the hypothesis was not tenable). No correction for multiple testing was applied. As a sensitivity analysis, the analyses were performed with p‐tau217 values residualized based on run number, to assess if there was a significant run‐induced clustering effect. As residualizing based on run number had no effect on the statistical output, results without run number residualization are presented. Run number was previously determined not to have a significant influence on concentration of NfL, GFAP, and Aβ (data not published).

#### 2.3.1 Parameters estimated in twin designs

Model specifications are described in detail in the Methods section in supporting information. Briefly, the classical twin model relies on the fundamental assumption that monozygotic (MZ) twins share 100% of their additive genetic variance, while dizygotic (DZ) twins share, on average, 50%. This difference in genetic relatedness between MZ twins and DZ twins is leveraged to estimate the genetic and environmental contributions to variance in a trait. In the univariate classical twin design, phenotypic variance can be decomposed into four components: additive genetic (A) variance, non‐additive genetic (D) variance, shared environmental (C) variance, and unshared environmental (E) variance.^34^ Shared environment is the environment all family members experience (contributing to their phenotypic covariance), while unshared environment is experienced by only one family member (not a source of phenotypic covariance). The latter also includes measurement error.

#### 2.3.2 Univariate extended twin design

**FIGURE 1:**
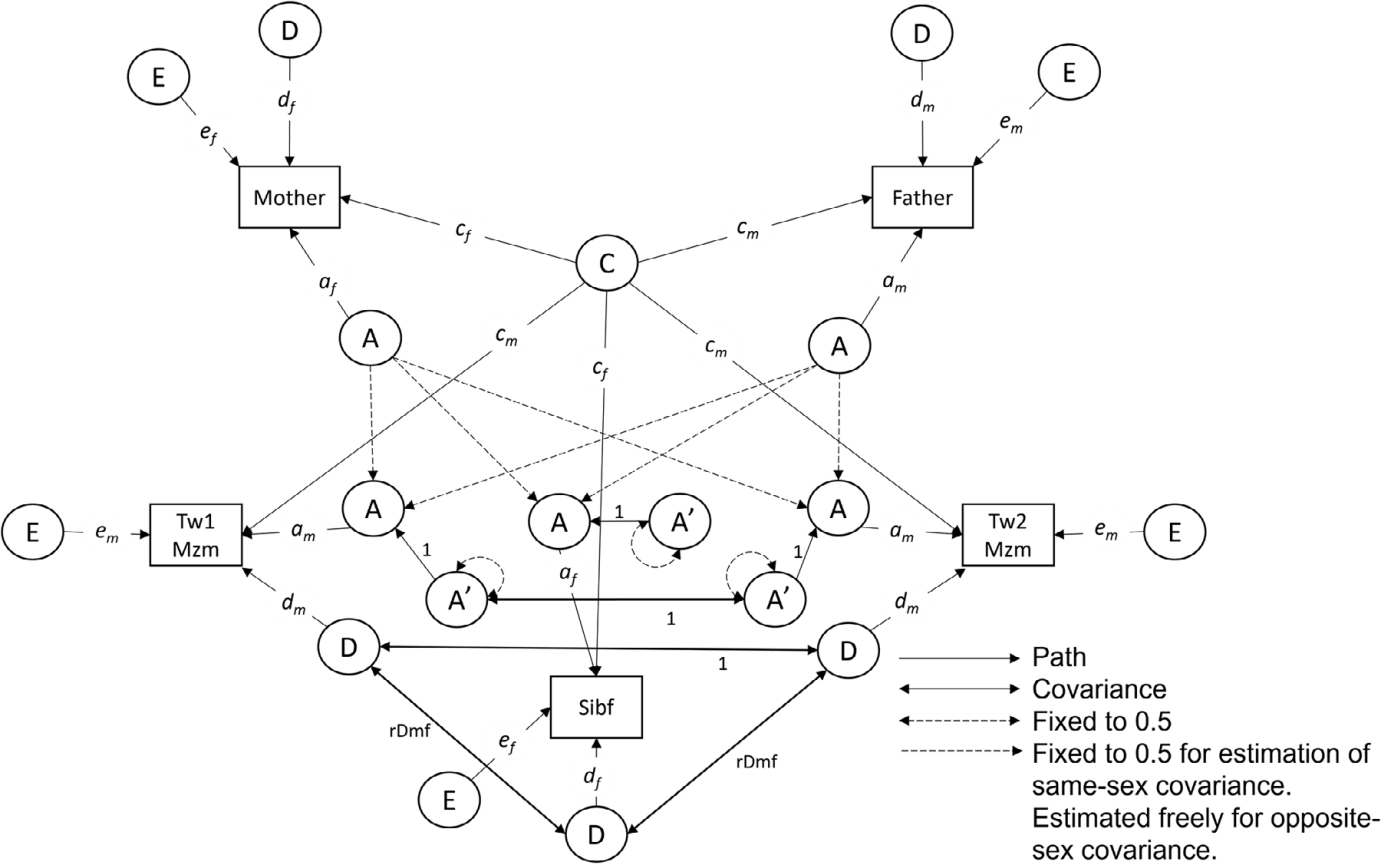
Example univariate extended twin model with sex differences for a family with two monozygotic male twins, a female non‐twin sibling, a mother and a father. A, additive genetic factors; A’, additive genetic factors residuals due to mendelian segregation; a*_f _* = effect of additive genetic factors in females; a*_m_*, effect of A in males; C, shared environmental factor; c*_f_*, effect of C in females; c*_m_*, effect of C in males; D, non‐additive genetic factors; d*_f_*, effect of non‐additive genetic factors in females; d*_m_*, effect of non‐additive genetic factors in males. E, unshared environmental factors; e*_f_*, effect of unshared environmental factors in females; e*_m_*, effect of unshared environmental factors in males; Mzm, monozygotic males; rDmf, free covariance parameter between the non‐additive genetic factors in males and females; Sibf, female non‐twin siblings; Tw1/2, twin 1/2.

The univariate extended (i.e., including parents and siblings) twin design was used to detect both quantitative and qualitative contributions to variance in p‐tau217 concentration by sex (Figure 1). Sex differences can be quantitative or qualitative.^43^ Quantitative sex differences are differences in the magnitude of genetic and environmental influences operating in both males and females. Qualitative sex differences are differences in actual genetic and environmental factors influencing the phenotype, that is, sex‐specific genetic and environmental influences. Qualitative sex differences can arise from different genes or environments being involved in males and females. Due to modeling limitations, it was not possible for us to test for both genetic and environmental qualitative sex differences. Based on family correlations of the biomarkers, we opted to test for qualitative genetic sex differences. To test for sex differences, twins, siblings, and their parents were categorized on the basis of the zygosity and sex of the twins (MZ males, MZ females, DZ males, DZ females, DZ opposite sex; see Methods in supporting information). The inclusion of opposite‐sex DZ twins is crucial for disentangling genetic and shared environmental effects that may operate differently in males and females.^44^

Considering age is a major risk factor for AD, we conducted an age moderation analysis. This analysis allowed us to determine whether genetic and/or environmental effects became stronger or weaker over age. In age moderation models, specific age moderation terms are modeled for all genetic (additive and non‐additive) and environmental (shared and unshared) components of the models. We dropped these genetic and environmental specific age moderation terms from the model one at a time, and tested the age moderation using the likelihood ratio test. We fitted an age moderation model separately in males and females to account for potential sex effects in the moderation of age.[Fig alz70906-fig-0002]

#### 2.3.3 Multivariate extended twin design

To determine whether the correlation between two phenotypes is due to shared genetic and/or shared environmental factors, the covariance between these two phenotypes is decomposed into the covariance between their genetic factors (A and D) and the covariance between their environmental factors (C and E).

**FIGURE 2 alz70906-fig-0002:**
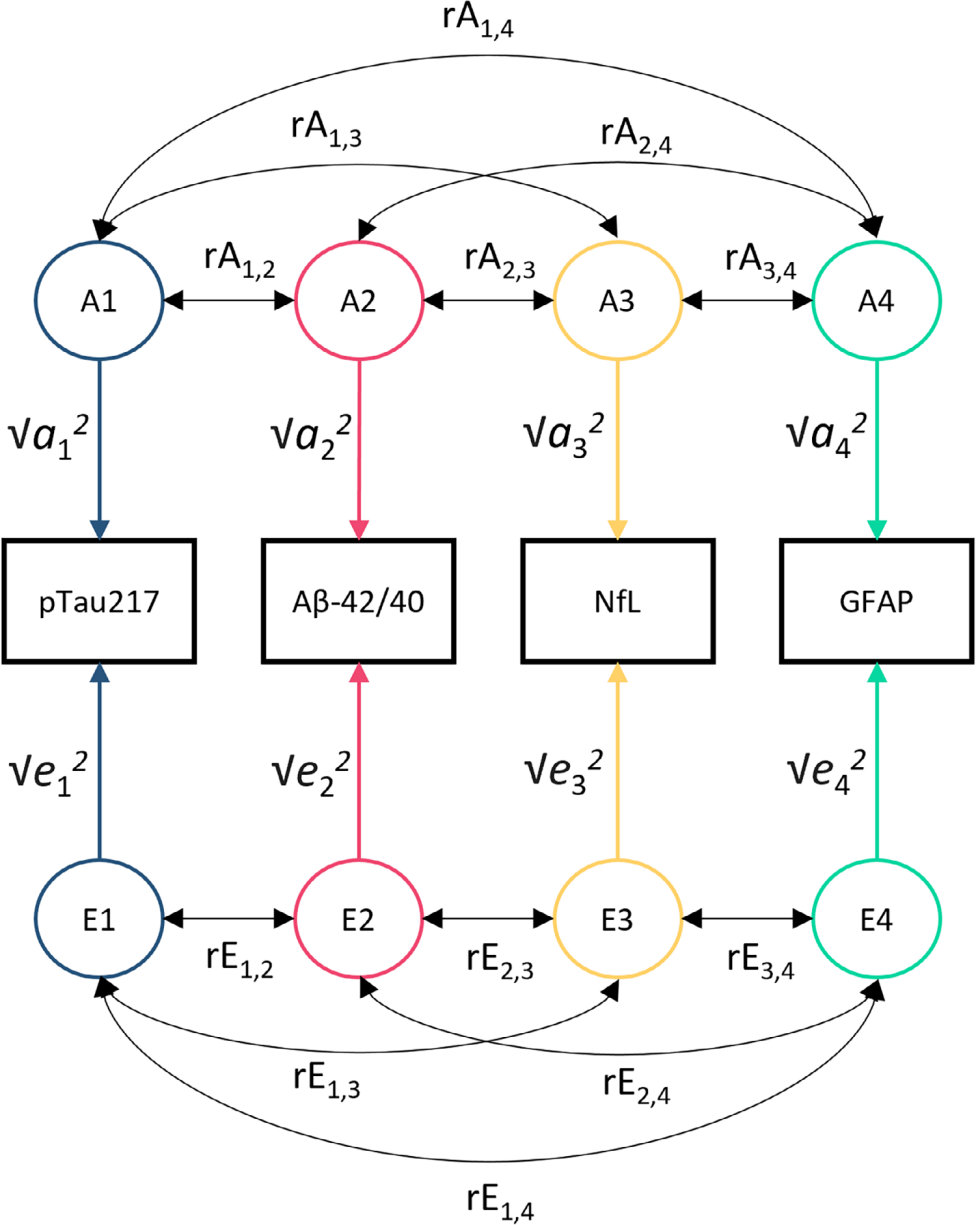
Multivariate AE model for the four standardized biomarkers within one family member. The model was fit separately in males and females. Variance of biomarker X = a_x_^2^ + e_x_^2^ = 1. Covariance between biomarker X and biomarker Y = a_x_*rA_x,y_*a_y_ + e_x_*rE_x,y_*e_y_. A, additive genetic factors; Aβ, amyloid beta; E, unshared environmental factors; GFAP, glial fibrillary acidic protein; *N*, number; NfL, neurofilament light chain; p‐tau, phosphorylated tau; √a_x_^2^, effect of A on biomarker X; √e_x_^2^, effect of unshared environmental factors on biomarker X; rA_x,y_, correlation between genetic factors influencing biomarker X and biomarker Y; rE_x,y_, correlation between environmental factors influencing biomarker X and biomarker Y.

The contributions of genetic and environmental factors to the phenotypic variance in NfL, GFAP, and Aβ42/40 concentrations have previously been studied in this cohort.^32^ Additive genetic (A) and unshared environmental (E) factors were found to contribute to NfL and Aβ42/40 variance, while additive (A), non‐additive genetic (D), and unshared environmental (E) effects were found for GFAP variance. No sex differences were found for these biomarkers. A multivariate extended twin design was fit to test for correlations between A, D, C, and E factors contributing to the variance of p‐tau217, NfL, GFAP, and Aβ42/40 (Figure 2).^45^ Due to the detection of sex differences in the p‐tau217 univariate model, the multivariate models were fit in males and females separately. The female group included female twins (MZ females, DZ females, and the female twin of opposite sex twin pairs), their female siblings, and their mothers. The male group included male twins (MZ males, DZ males, and the male twin of opposite sex twin pairs), their male siblings, and their fathers.

The phenotypic covariance between all four biomarkers was decomposed into A, D, C, and E covariance matrices using the Cholesky decomposition parameterization,^33^ allowing for the estimation of variance components and covariances for all biomarkers simultaneously. The estimates from the covariance matrices were standardized, giving the genetic and environmental correlations between the different biomarkers. These genetic correlations indicate the extent to which the same underlying genes influence the variability of the two biomarkers, while environmental correlations indicate the extent to which environmental factors contribute to their covariation. Note that unshared environmental influences do not contribute to the phenotypic covariance among family members, but can contribute to the within‐person covariance between phenotypes. To test the hypothesis that the genetic or environmental covariance is zero, we conducted a likelihood ratio test.

## 3 RESULTS

The number of complete twin pairs, age, and biomarker concentrations are reported in Table 1. The proportion of participants who are Aβ+ based on their p‐tau217 concentrations was determined using the cutoff defined by Schindler et al.^46^ Mean concentrations of p‐tau217 by sex and age are shown in Figure S2 in supporting information.

**TABLE 1:** Family demographics.

	N (Complete pairs)	Age Mean (SD) [range]	p‐tau217 concentration Mean (SD)	Aβ42/40 ratio Mean (SD)	NfL concentration Mean (SD)	GFAP ratio Mean (SD)	Proportion Aβ+ *N* (%)
**Monozygotic twins**	1573 (637)	42 (11) [30–90]	0.0269 (0.0204)	0.0700 (0.0230)	7.71 (5.40)	57.7 (30.6)	47 (3.15%)
Male pairs	469 (182)	41 (12) [30–86]	0.0309 (0.0181)	0.0725 (0.0218)	7.97 (5.98)	53.3 (28.8)	20 (4.62%)
Female pairs	1104 (455)	42 (11) [30–90]	0.0252 (0.0211)	0.0690 (0.0234)	7.61 (5.13	59.5 (31.2)	27 (2.55%)
**Dizygotic twins**	1266 (432)	39 (10) [30–89]	0.0268 (0.0196)	0.0683 (0.0156)	7.54 (14.7)	54.3 (49.5)	32 (2.53%)
Male pair	228 (72)	38 (11) [30–79]	0.0306 (0.0172)	0.0679 (0.0139)	7.13 (6.27)	52.2 (57.6)	9 (3.96%)
Female pairs	503 (195)	40 (10) [30–86]	0.0251 (0.0198)	0.0677 (0.0158)	7.32 (5.37)	57.7 (36.7)	12 (2.39%)
Opposite sex pairs	535 (165)	38 (9) [30–89]	0.0269 (0.0201)	0.0690 (0.0161)	7.92 (21.6)	52.0 (55.8)	11 (2.06%)
**Siblings**	577	51 (10) [40–89]	0.0302 (0.0255)	0.0668 (0.0150)	9.76 (8.22)	68.1 (69.6)	18 (4.69%)
Males	197	53 (11) [40–84]	0.0335 (0.0216)	0.0662 (0.0139)	10.7 (7.99)	61.7 (31.0)	9 (6.16%)
Females	380	50 (9) [40–89]	0.0282 (0.0275)	0.0672 (0.0157)	9.21 (8.34)	72.0 (84.8)	9 (3.78%)
**Parents**	3079 (921)	58 (8) [40–98]	0.0331 (0.0239)	0.0658 (0.0171)	11.8 (9.78)	77.6 (63.4)	180 (6.73%)
Fathers	1182	60 (6) [40–89]	0.0360 (0.0255)	0.0647 (0.0146)	13.4 (11.3)	77.6 (50.8)	89 (8.57%)
Mothers	1897	56 (8) [40–98]	0.0312 (0.0225)	0.0665 (0.0185)	10.8 (8.52)	77.5 (70.3)	91 (5.56%)
**Total**	6495	50 (13) [30–98]	0.0308 (0.0242)	0.0672 (0.0182)	10.2 (11.3)	69.3 (56.1)	277 (5.00%)

*Note*: The proportion of amyloid positive (Aβ+) participants is based on their p‐tau217 concentrations and the cutoff defined by Schindler et al.^46^

Abbreviations: Aβ, amyloid beta; GFAP, glial fibrillary acidic protein; *N*, number; NfL, neurofilament light chain; p‐tau, phosphorylated tau; SD, standard deviation.

### 3.1 Univariate extended twin design—quantitative and qualitative sex differences

**FIGURE 3:**
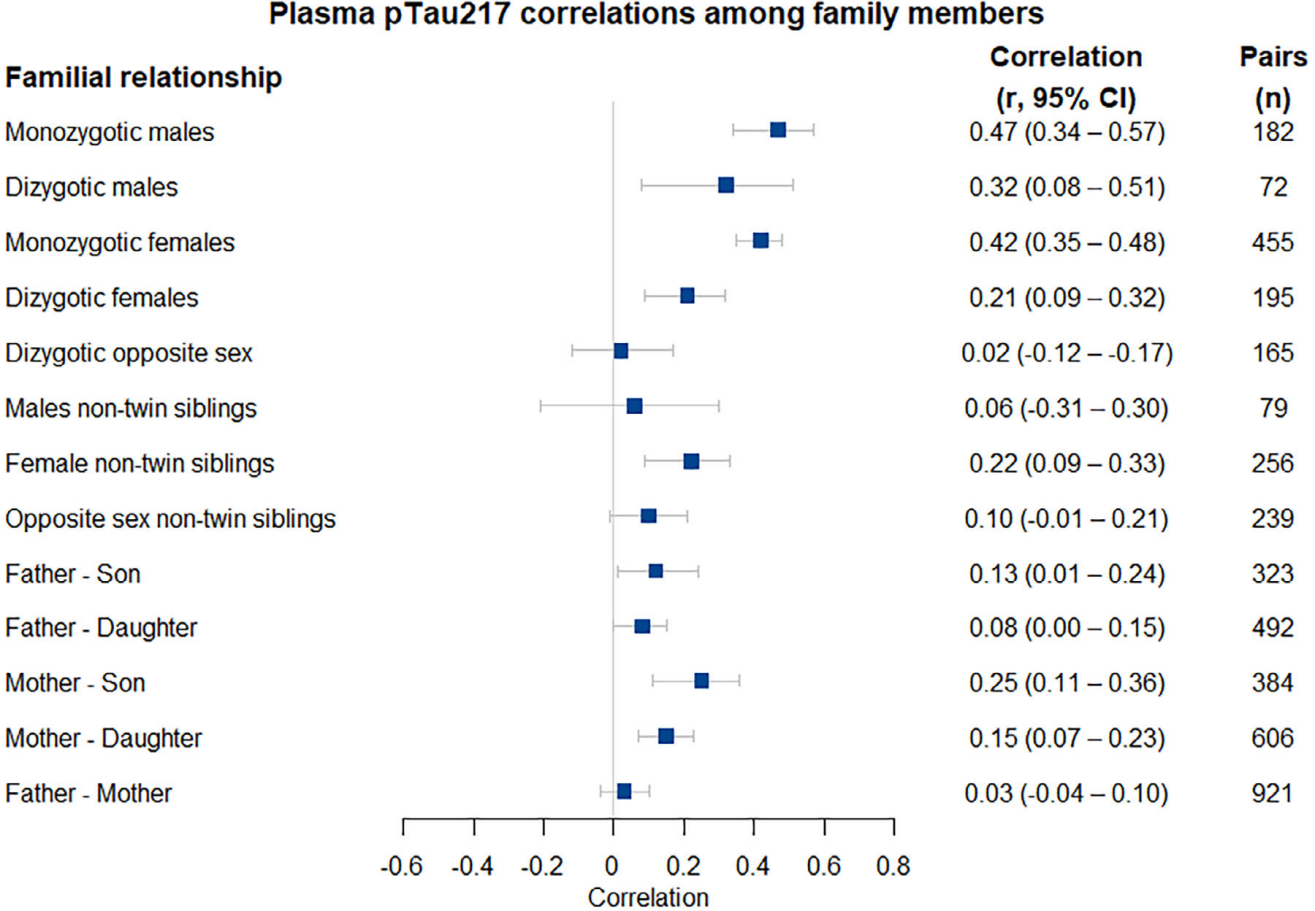
Phenotypic correlation of plasma p‐tau217 between participants sharing different familiar relationships. All correlations between family members were adjusted for age, sex, and age by sex effects. Male non‐twin siblings refers to correlations between male twins and their male non‐twin sibling, or between two male non‐twin siblings. Female non‐twin siblings refers to correlations between female twins and their female non‐twin sibling, or between two female non‐twin siblings. Opposite sex non‐twin siblings refers to correlations between male twins and their female non‐twin sibling, female twins and their male non‐twin sibling, or between a male and a female non‐twin siblings. Pairs indicates the number of pairs the reported correlation was based on. If the 95% confidence interval (95%) includes 0, then the correlation is not significant. Stronger correlations typically indicate that individuals of a given familial relationship share a larger amount of genetics and/or environment that effects p‐tau217 concentrations. Correlations being stronger for individuals who share a higher proportion of their genetic (e.g., monozygotic twins compared to dizygotic twins, father–son compared to father–mother) indicates that genetic effects play a role in variation in p‐tau217 concentrations. CI, confidence interval; p‐tau, phosphorylated tau.

The results of the testing of assumptions about equal means and variances across family roles and sex are presented in the Methods (supporting information) and Table S1 in supporting information. Phenotypic correlations between family members are presented in Figure 3.

No evidence of shared environment (C) or non‐additive genetic (D) effects was found for either sex. The relative contribution of additive genetic (A) to variability in p‐tau217 concentration was estimated at 42% for males and 41% for females, indicating minimal quantitative sex differences in the overall magnitude of genetic influence. However, the correlation of 0.23 (95% CI: 0.10–0.37) between the additive genetic factors in males and females was indicative of strong qualitative sex differences. Were the qualitative sex differences absent, this correlation would be 0.50. The detailed model specifications and complete results are summarized in Table 2.

**TABLE 2:** Standardized estimates of the ADCE extended twin design (95% CIs in brackets).

	SA	SD	SC	SE	rAmf	rDmf	df	ΔLL	AIC	*p* value
ADCE with qualitative and quantitative sex differences										
Male	0.12 [0–0.46]	0.30 [0–0.50]	0.06 [0–0.21]	0.53 [0.43–0.65]	1 [‐1–1]	−0.09 [‐1–1]	5802		15484	
Female	0 [0–0.42]	0.28 [0–0.38]	0.14 [0–0.21]	0.58 [0.51–0.64]						
ADE with qualitative and quantitative sex differences										
Male	0.24 [0.02–0.46]	0.24 [0–0.49]	–	0.53 [0.43–0.65]	0.38 [0.13–1]	−0.14 [‐1–1]	5804 (Δdf = 2)	1.13	15482	0.57
Female	0.30 [0.16–0.44]	0.13 [0–0.29]	–	0.57 [0.51–0.64]						
AE with qualitative and quantitative sex differences										
Male	0.42 [0.30–0.52]	–	–	0.58 [0.48–0.70]	0.23 [0.10–0.37]	–	5807 (Δdf = 5)	7.15	15482	0.21
Female	0.41 [0.34–0.47]	–	–	0.59 [0.53–0.66]						
AE with quantitative sex differences										
Male	0.30 [0.18–0.42]	–	–	0.70 [0.58–0.82]	0.50	–	5808 (Δdf = 1)	12.6	15492	3.8e‐04
Female	0.38 [0.32–0.45]	–	–	0.61 [0.55–0.68]						
AE without sex differences	0.36 [0.31–0.41	–	–	0.64 [0.59–0.69]	–	–	5810 (Δdf = 3)	132	15607	2.3e‐28

*Note*: If there are no sex differences, rA = 0.5 and rD = 0.25.

Abbreviations: A, additive genetic factors; C, shared environmental factor; D, non‐additive genetic factor; E, unshared environmental factors; ADCE, xxxxx; AIC, Akaike information criterion; CI, confidence interval; rAmf, correlation of A factors between males and females (father–daughter, mother–son, opposite sex siblings); rDmf, correlation of D factors between males and females (opposite sex siblings); SA, standardized estimate of additive genetic influences; SC, standardized estimate of shared environmental influences; SD, standardized estimate of non‐additive genetic influences; SE, standardized estimate of unshared environmental influences.

Based on these results, the age moderation models were fit in males and females separately, and only additive genetic effects and unshared environmental effects were modeled. In males, we found that there was no age moderation effect on additive genetic effects (*p* value = 0.19), but that there was a significant age moderation effect on unshared environmental effects (*p* value = 2.84e‐07). Specifically, as age increases, so does the proportion of variance that is explained by unshared environmental effects (Table S2 and Figure S3A in supporting information). In females, the age moderation effect on additive genetic effects was not significant (*p* value = 0.77), and neither was the age moderation effect on unshared environmental effects (*p* value = 0.27). However, the model that excluded the age moderation effects on both the additive genetic effect and the unshared environmental effect was a worse fit (*p* value = 0.003). We therefore report the results of the model with age moderation effects on both additive genetic effects and unshared environmental effects, which indicate an increased proportion of variance being explained by additive genetic effects with older age, paired with a decreased proportion of variance being explained by unshared environmental effects (Table S2, Figure S3B).

### 3.2 Multivariate extended twin design—genetic and environmental correlations between biomarkers

The aim of the multivariate extended twin design was to determine whether the genetic and environmental factors influencing plasma concentrations of p‐tau217, Aβ42/40, NfL, and GFAP were correlated. If two biomarkers are correlated with their genetic and environmental factors, it supports the hypothesis that they may be involved in a common pathway. As in the univariate analysis, neither non‐additive genetic effect nor shared environmental effects were found to contribute to either p‐tau217, NfL, GFAP, or Aβ42/40 in males or females (*p* value = 0.52).

#### 3.2.1 Males

The results of the multivariate model examining genetic and environmental correlations among p‐tau217, NfL, GFAP, and Aβ42/40 in males are presented below. Low to moderate phenotypic correlations were found between p‐tau217 and NfL (*r* = 0.23, 95% CI: 0.19–0.26), p‐tau217 and GFAP (*r* = 0.25, 95% CI: 0.21–0.29), and NfL and GFAP (*r* = 0.36, 95% CI: 0.32–0.39; Figure 4A). A low negative phenotypic correlation of −0.07 (95% CI: −0.11 to −0.03) was found between p‐tau217 and Aβ42/40. The phenotypic correlations between the Aβ42/40 ratio and both NfL and GFAP were not significant (*r* = 0, 95% CI: –0.04 to 0.04 for both).

FIGURE 4Phenotypic, genetic, and environmental correlations among p‐tau217, NfL, GFAP, and the Aβ42/40 ratio. A, correlations in males; Aβ, amyloid beta; B, correlations in females; GFAP, glial fibrillary acidic protein; NfL, neurofilament light chain; p‐tau, phosphorylated tau.

**Figure:**
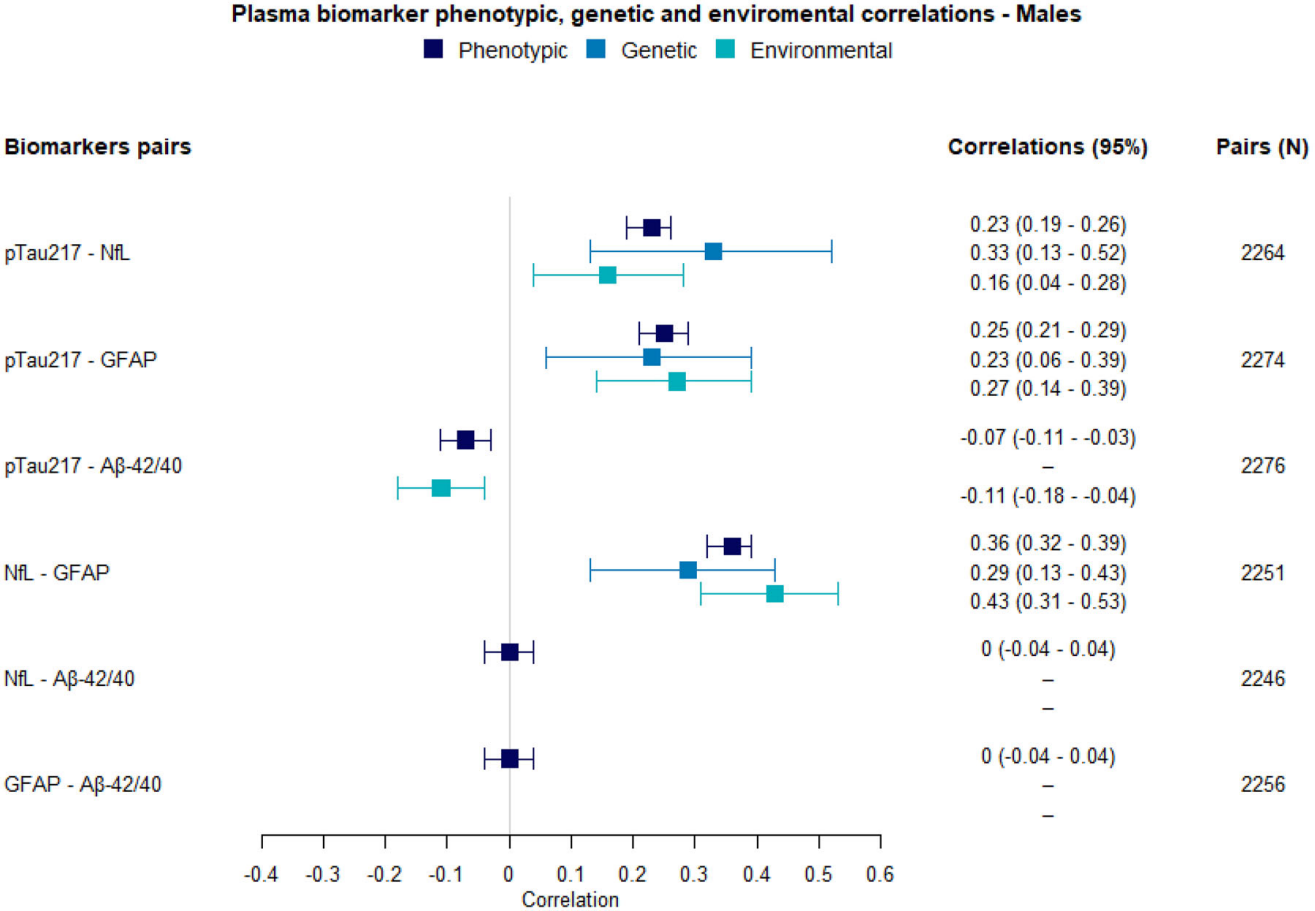

**Figure:**
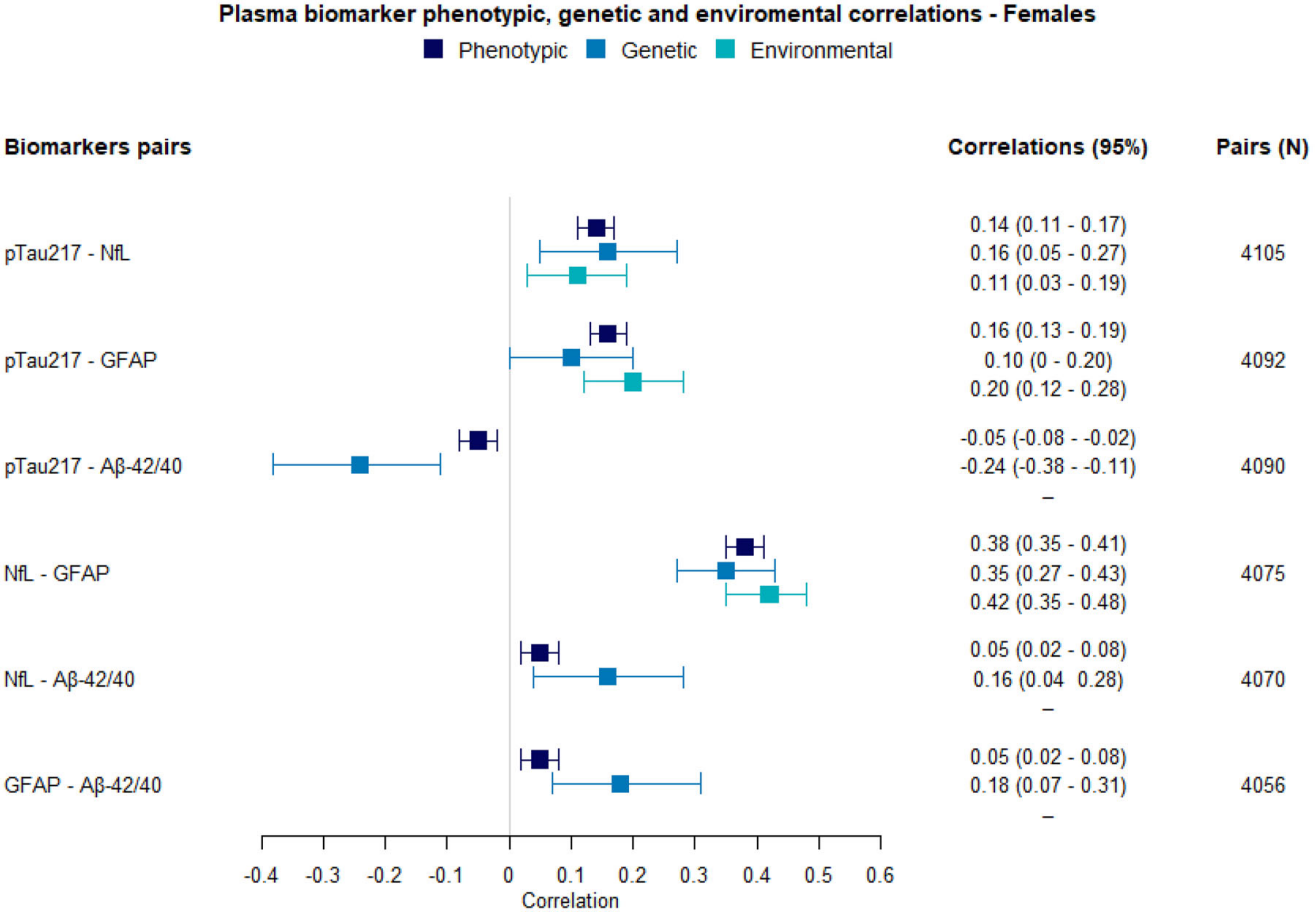

Figure S4A in supporting information illustrates the results of the multivariate model in males. Significant genetic (rA) and environmental (rE) correlations were found between p‐tau217 and NfL, p‐tau217, and GFAP, and NfL and GFAP. For p‐au217 and NfL, the genetic correlation was 0.33 (95% CI: 0.13–0.52), and explained 60% of the phenotypic correlation, while the environmental correlation was 0.16 (95% CI: 0.04–0.28) and explained the remaining 40%. For p‐tau217 and GFAP, the genetic correlation of 0.23 (95% CI: 0.06–0.39) explained 45% of the phenotypic correlation, and the environmental correlation of 0.27 (95% CI: 0.14–0.39) explained the remaining 55% variance. The genetic correlation between NfL and GFAP was 0.29 (95% CI: 0.13–0.43) and explained 40% of the phenotypic correlation, while the environmental correlation was 0.43 (95% CI: 0.31–0.53) and explained the other 60%. Additionally, a low negative environmental correlation of −0.11 (95% CI: −0.28 to −0.04) was found between p‐tau217 and the Aβ42/40 ratio, while no genetic correlation was observed. No genetic or environmental correlations were found between the Aβ42/40 ratio and NfL nor GFAP. Notably, the negative association between p‐tau217 and Aβ42/40 in males appears to be driven by common environmental influences. Model details are reported in Table S3 in supporting information.

#### 3.2.2 Females

The results of the multivariate model examining genetic and environmental correlations among p‐tau217, NfL, GFAP, and Aβ42/40 in females are as follows. Phenotypic correlations between p‐tau217 and NfL (*r* = 0.14, 95% CI: 0.11–0.17) and p‐tau217 and GFAP (*r* = 0.16, 95% CI: 0.13–0.19) were overall weaker than in males (Figure 4B). The phenotypic correlation between p‐tau217 and the Aβ42/40 (*r* = –0.05, 95% CI: −0.08 to −0.02) and NfL and GFAP (*r* = 0.38, 95% CI: 0.35–0.41) was found to be similar in females and males. Additionally, low phenotypic correlations were found between the Aβ42/40 ratio and NfL and GFAP (*r* = 0.05, 95% CI: 0.02–0.08 for both), which were zero in males.

Figure S4B illustrates the results of the multivariate model in females. As was the case with males, significant genetic and environmental correlations were found between p‐tau217 and NfL, p‐tau217 and GFAP, and NfL and GFAP. The genetic correlation between p‐tau217 and NfL was 0.16 (95%: 0.05–0.27), explaining 53% of the phenotypic correlation, and the environmental correlation was 0.11 (95% CI: 0.03–0.19), explaining 47% of the phenotypic correlation. Between p‐tau217 and GFAP, the genetic correlation was 0.10 (95% CI: 0–0.20), explaining 34% of the phenotypic correlation, and the environmental correlation was 0.20 (95% CI: 0.12–0.28), explaining the rest. For NfL and GFAP, the genetic correlation was 0.35 (95% CI: 0.27–0.43), explaining 49% of the phenotypic correlation, and the remaining 51% was explained by the environmental correlation of 0.42 (95% CI: 0.35–0.48).

Additionally, a genetic correlation of −0.24 (95% CI: −0.38 to –0.11) was found between p‐tau217 and the Aβ42/40 ratio, as well as a genetic correlation of 0.16 (95% CI: 0.04–0.28) between NfL and the Aβ42/40 ratio and a genetic correlation of 0.18 (95% CI: –0.07 to 0.31) between GFAP and Aβ42/40. No environmental correlations were found for Aβ42/40 with NfL, GFAP, or p‐tau217. Notably, the negative association between p‐tau217 and Aβ42/40 in females appears to be driven by shared genetic influences, contrasting with the environmental influence observed in males. Model details are reported in Table S4 in supporting information.

## 4 DISCUSSION

This study aimed to quantify the relative genetic and environmental contributions to variance in plasma p‐tau217 concentrations. Genetic contributions were moderate in males (42%) and females (41%) and were partially due to sex‐specific genetic effects. We also found age moderation of the genetic effects in females and environmental effects in both sexes. Specifically, we observe increased environmental contributions with age in males and a decrease in females. These estimates are lower than the previous estimate of 56%,^17^ which may be due to our inclusion of parents and siblings of the twins, and our larger cohort most likely being more heterogeneous. Additionally, we report a correlation between the genetic and environmental factors influencing p‐tau217, NfL, and GFAP in both males and females. A genetic correlation was also found for p‐tau217 and Aβ42/40 in females, while environmental factors were correlated for p‐tau217 and Aβ42/40 in males.

Sex differences have previously been reported regarding the association of plasma p‐tau217 and p‐tau181 with AD clinical outcomes. One study found that in cognitively unimpaired (CU) individuals, elevated plasma p‐tau217 concentrations correlate with a steeper decline in cognition and more pronounced medial temporal lobe atrophy in females only.^18^ Another study reported that at the mild cognitive impairment stage, plasma p‐tau181 concentrations were more strongly associated with brain glucose metabolism, cognitive decline, and brain Aβ deposition in females than males.^18^, ^19^ In individuals with amyloid pathology, plasma p‐tau181 was a stronger predictor of conversion to dementia in females than males.^19^ Sex differences in genetic risk for AD have also been reported, with multiple genes having female‐specific or male‐specific effects.^47^ Our finding of sex‐specific genetic effects on p‐tau217 concentration may relate to these AD‐specific sex differences, indicating that the female‐specific genetic factors involved in p‐tau217 concentrations are more strongly associated with AD risk and processes than male‐specific genetic factors. However, because of our inclusion criteria of lack of cognitive impairment, our cohort only consists of an unknown mix of participants without AD pathology and participants with pre‐symptomatic AD. Therefore, we cannot exclude that our findings of sex‐specific genetic effects are not directly related to pathology, and that previously reported sex differences in AD‐related processes are mitigated by other genetic or environmental factors.

We also report age moderation on genetic effects in females, and on environmental effects in both males and females. In males, we observe that environmental influences on plasma p‐tau217 concentrations become exponentially stronger over age. In females, we observe a much less steep decrease in environmental influences over age, as well as a small increase in genetic influences. The stark difference in age moderation on environmental influences between the sexes could be due to a sex by environment interaction effect, meaning that the same environment exerts different effects in males and females, or it could be a reflection of males and females being exposed to different environments. For example, men are 40% more likely to suffer from traumatic brain injuries,^48^ which have been associated with elevated p‐tau217.^49^

Genetic and environmental correlations were found between p‐tau217 and other AD plasma biomarkers. Low to moderate genetic and environmental correlations were found for p‐tau217 with NfL and GFAP, suggesting these three biomarkers may be part of a common biological pathway.^50^ A low negative correlation was also found between p‐tau217 and the Aβ42/40 ratio. In males, it was explained solely by correlating environmental factors, while in females, it was explained by correlating genetic factors only. Out of the four biomarkers investigated in this study, p‐tau217 and the Aβ42/40 are the two that are most specific to AD pathology.^51^ The overall low correlation between the two indicates that, in individuals without clinical AD pathology, they may reflect separate biological processes. However, it cannot be excluded that these two biomarkers are also involved in shared AD‐specific biological processes, which could not be separated from non‐AD processes, as we did not formally assess our participants for AD pathology. Our study has several limitations. First, as we wanted to investigate genetic and environmental effects on p‐tau217 concentrations independent of AD pathology, we chose to use data from a relatively young and CU cohort (mean age of 50, while AD‐related complaints usually begin after age 65^52^). However, as our participants did not undergo rigorous testing to rule out AD, we cannot guarantee that all participants were free of AD pathology. Our results may also incompletely generalize to a population with clinical AD. Second, we report a significant age moderation effect on the relative contributions of genetic and environmental factors on p‐tau217, meaning the genetic and environmental estimates we report are not static and likely to be different when investigating different age groups. In the twin design, the unique environmental contribution estimates may be inflated due to measurement error; however, the coefficient of variation percentage for all biomarkers was quite low (< 10%), indicating low variation in measurement.

This study has benefited from the following strengths. Our large cohort allowed us to detect significant sex differences in p‐tau217 expression. As these differences are relatively small, they might not be detectable in smaller samples. Finally, our inclusion of both parents and siblings of twins enabled us to simultaneously estimate additive genetic, non‐additive genetic, shared environment, and unshared environment influences, which is not possible with twins only.

Taken together, our findings indicate that the genetic contributions to plasma p‐tau217 concentrations are moderate and partially due to sex‐specific genetic influences. We report correlations among p‐tau217, NfL, and GFAP, which supports the hypothesis that these biomarkers are part of shared biological processes. Additionally, we report a weak correlation between p‐tau217 and Aβ42/40, indicating potential divergent pathways for these biomarkers prior to clinical onset of AD. Finally, environmental factors also strongly contribute to variance in pTau217 concentration. Additional research on environmental factors and their effects on p‐tau217 concentrations may further improve the interpretation of p‐tau217 concentrations.

## AUTHOR CONTRIBUTIONS

Rebecca Z. Rousset participated in the measurement of samples, defining the project outline, carrying out the statistical analysis, interpretation of results, and writing of the manuscript. Conor V. Dolan participated in the statistical analysis, interpretation of results, and writing of the manuscript. David H. Wilson provided input on the sample measurement process and results interpretation. Lisanne in ‘t Veld coordinated and participated in the measurement of samples. Lannie Ligthart participated in the preparation and interpretation of the data. Charlotte E. Teunissen participated in defining the project outline and contextualizing of results. Eco J. C. de Geus participated in defining the project outline, interpretation of results, and writing of the manuscript. Anouk den Braber participated in defining the project outline, interpretation of results, and writing of the manuscript.

## CONFLICT OF INTEREST STATEMENT

Charlotte E. Teunissen has research contracts with Acumen, ADx Neurosciences, AC‐Immune, Alamar, Aribio, Axon Neurosciences, Beckman‐Coulter, BioConnect, Bioorchestra, Brainstorm Therapeutics, Celgene, Cognition Therapeutics, EIP Pharma, Eisai, Eli Lilly, Fujirebio, Instant Nano Biosensors, Novo Nordisk, Olink, PeopleBio, Quanterix, Roche, Toyama, Vivoryon. She is editor in chief of *Alzheimer Research and Therapy*, and serves on editorial boards of Medidact Neurologie/Springer, and *Neurology: Neuroimmunology & Neuroinflammation*. She had consultancy/speaker contracts for Eli Lilly, Merck, Novo Nordisk, Olink, and Roche. David H. Wilson is an employee of Quanterix Corp. Rebecca Z. Rousset, Conor V. Dolan, Lisanne in ‘t Veld, Lannie Ligthart, Eco J. C. de Geus, and Anouk den Braber have nothing to disclose. Author disclosures are available in the supporting information.

## CONSENT STATEMENT

All participants gave consent during the biobank collection to have their samples and information used in epidemiological research.

## Supporting information



Supporting information

Supporting information
