# Supplementary material for: Genetic and environmental contributions to variation in plasma phosphorylated tau 217
Source: Alzheimers Dement. 2025 Nov 13;21(11):e70906. doi: 10.1002/alz.70906 (PMC12614086; doi:10.1002/alz.70906)
Supplement: Supplementary file 2 — Supporting information [file ALZ-21-e70906-s002.docx]

**Supplemental Online Content**

Rebecca Z. Rousset, Eco J.C. de Geus, Conor V. Dolan, David H. Wilson, Lisanne in ‘t Veld, Lannie Ligthart, Charlotte E. Teunissen, Anouk den Braber

**eMethods**

**eFigure 1.** Bland-Altman plot of the concentrations of pTau217 samples measured in duplicate.

**eFigure 2.** Raw pTau217 concentration plotted by sex over age.

**eTable 1.** Saturated model with sex differences for pTau217.

**eTable 2.** Summary of the age moderation model in males and females.

**eFigure 3.** Age moderation model in males and females.

**eFigure 4.** Path diagram of the multivariate extended design in males and females.

**eTable 3.** Summary of the multivariate model in males.

**eTable 4.** Summary of the multivariate model in females.

**eMethods**

Twin Designs

Twin designs refer to statistical approaches specifically designed to use data from twins. In the classical twin model, the difference in genetic relatedness between twins (monozygotic, or identical twins, and dizygotic, or fraternal twins) is used to estimate the genetic and environmental contributions to variance in a trait. In the univariate classical twin design, phenotypic variance can be decomposed into 4 components: additive genetic (A) variance, non-additive genetic (D) variance, shared environmental (C) variance and unshared environmental (E) variance. Given a locus with alleles B and b, the A variance is the variance explained by the linear regression of the phenotype of the locus coded as 0 (bb), 1 (bB or Bb) and 2 (BB). The D variance is due the departure of linearity of the relationship between the locus (coded 0,1,2) and the phenotype (1). The C variance is due to environmental influences on the phenotype which are shared by the family members (exposure to pollution, diet, etc). The E variance is due to environmental effects that are not shared by family member, i.e., are unique to an individual. Variance attributable to measurement errors is included in E variance.

Monozygotic (MZ) twins share 100% of their alleles, meaning they correlate 1 on A and D factors. Dizygotic (DZ) twins on average share 50% of their alleles, meaning they correlate about 0.50 on A factors and 0.25 on D factors, because D variance attributable to a given locus is shared only if the twins are genetically identical at the locus. This is expected to be the case in 25% of the DZ twins, hence the correlation of 0.25. Twin-siblings and sibling-sibling are the same as DZ twins in terms of correlation on A and D factors. Parent-offspring share 50% of their alleles, and thus correlate 0.5 on A factors. They do not correlate on D factors, because they cannot share more than one allele (offspring inherit one allele from each parent at a given locus). By definition, all family members correlate 1 on C factors, because these concern shared influences, and 0 on E factors, because these concern unshared influences. In the classical twin model (with or without additional siblings), only three of the four variance components can be estimated (2), i.e., the model fitted is either an ACE or an ADE model. However, adding the parents to the design allows for the estimation of all variance components (33), with the caveats that 1) the shared environment reflects only the environment shared when offspring and parents shared a household, and 2) gene-by-age and environment-by-age interactions are assumed to be absent; that is, it is assumed that the genetic and environment contributions (both in terms of which genes/environment exert an influence and the magnitude of that influence) do not change with age.

Sex differences in the Univariate Extended Twin Design

In the model with both quantitative and qualitative sex differences, the effects of A, D, C, and E were estimated separately for males (m) and females (f) (am, af, dm, df, cm, cf, em, ef). Additionally, for opposite sex pairs, a correlation parameter was estimated freely between the male and female A factors (rAmf) (father-daughter, mother-son, opposite sex twins/siblings) and the male and female D factors (rDmf) (opposite sex twins/siblings only). If there are no qualitative sex-differences, the A and D factors represent the same genes in males and females, and thus rAmf = 0.5 and rDmf = 0.25. To test for qualitative sex differences, rAmf and rDmf were fixed to 0.5 and 0.25, respectively. If this worsens the model fit, there is evidence of qualitative sex differences. To test for quantitative sex differences, the effects of A, D, C, and E in males and females were equated (am = af, dm = df, cm = cf, and em = ef, given that rAmf = 0.5 and rDmf = 0.25). If this worsens the model fit, there is evidence for quantitative sex differences.

Saturated model

Assumptions about equal means and variances across birth order, MZ and DZ twins, twins and siblings, and parents and offspring were first tested. Means and variances of male offspring (male twins and male siblings) were not significantly different and could be equated (p-value = 0.17). The same was found for means and variance of female offspring (female twins and female siblings) (p-value = 0.17). The mean of the male offspring and the male parents (fathers) could not be equated (p-value = 0.006), however the variance could (p-value = 0.15). For female offspring and female parents (mothers), neither the mean (p-value < 0.001) nor the variance (p-value < 0.001) of the two groups could be equated. (eTable 1).


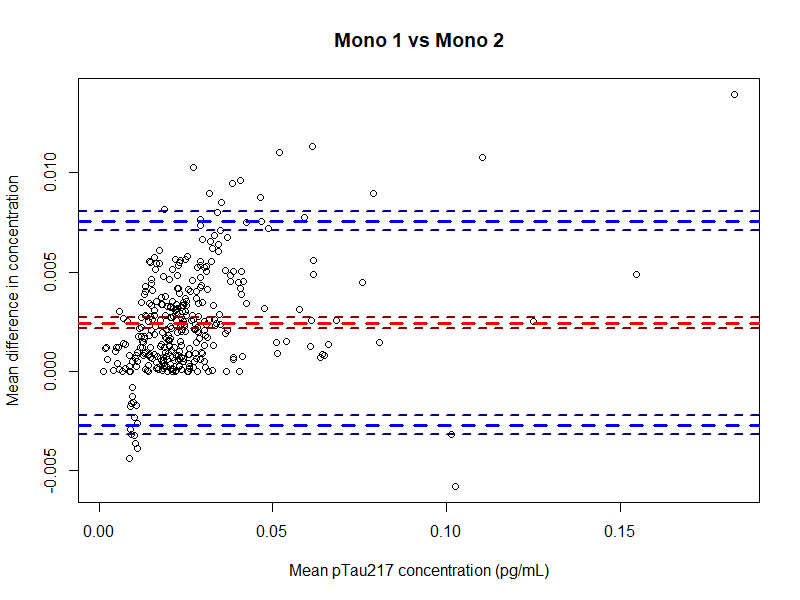


**eFigure 1. Bland-Altman plot of the concentrations of samples measured in duplicate.**

The mean difference of a sample’s first and second measurement is 0.0024 (95% Confidence interval: -0.0027 – 0.0076).

*
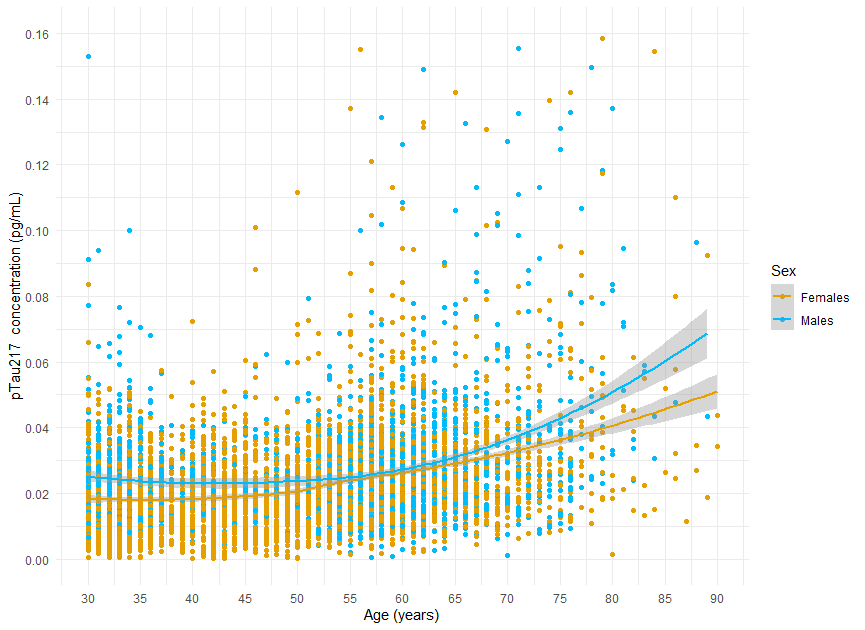
*

**eFigure 2. Raw pTau217 concentrations plotted by sex over age.**

| **eTable 1. Saturated model with sex differences for pTau217.** | | | | |
| --- | --- | --- | --- | --- |
| **Model** | **df** | **ΔLL** | **AIC** | **p-value** |
| Full saturated | 5772 |  | 15602 |  |
| Twin means constrained across birth order | 5776 | 3.11 | 15597 | 0.54 |
| Twin means and variances constrained across birth order | 5780 | 19.1 | 15605 | 0.01 |
| Twin means and variances constrained across birth order and zygosity | 5784 | 20.4 | 15599 | 0.06 |
| Twin means and variances constrained across birth order and zygosity and twins from same sex/opposite sex pairs | 5784 | 22.6 | 15593 | 0.12 |
| Siblings means constrained across birth order | 5790 | 24.3 | 15591 | 0.15 |
| Siblings means and variances constrained across birth order | 5792 | 25.4 | 15588 | 0.19 |
| Twins and siblings means constrained for same sex | 5794 | 26.0 | 15584 | 0.25 |
| Twins and siblings means and variances constrained for same sex | 5796 | 30.5 | 15585 | 0.17 |
| Male offspring and father means constrained | 5797 | 46.4 | 15599 | 0.01 |
| Male offspring and father variances constrained | 5797 | 32.2 | 15584 | 0.15 |
| Female offspring and mother means constrained | 5797 | 100 | 15653 | 5.4e-11 |
| Female offspring and mother variances constrained | 5797 | 60.1 | 15612 | 1.0e-04 |
| Offspring and parent means and variances constrained for same sex | 5800 | 147 | 15694 | 1.9e-41 |

| **eTable 2. Model with age moderation effect on genetic and environmental effects in males and females.** | | | | | |
| --- | --- | --- | --- | --- | --- |
| Sex | Model | df | ΔLL | AIC | p-value |
| Male | AE with age moderation on A and E | 1527 |  | 3582 |  |
|  | AE with age moderation on E | 1528 | 1.75 | 3582 | 0.19 |
|  | AE with age moderation on A | 1528 | 26.4 | 3607 | 2.84e-07 |
|  | AE without age moderation | 1529 | 39.9 | 3618 | 2.21e-09 |
| Female | AE with age moderation on A and E | 3213 |  | 9163 |  |
|  | AE with age moderation on E | 3214 | 0.09 | 9161 | 0.77 |
|  | AE with age moderation on A | 3214 | 1.24 | 9162 | 0.27 |
|  | AE without age moderation | 3215 | 11.9 | 9170 | 0.003 |


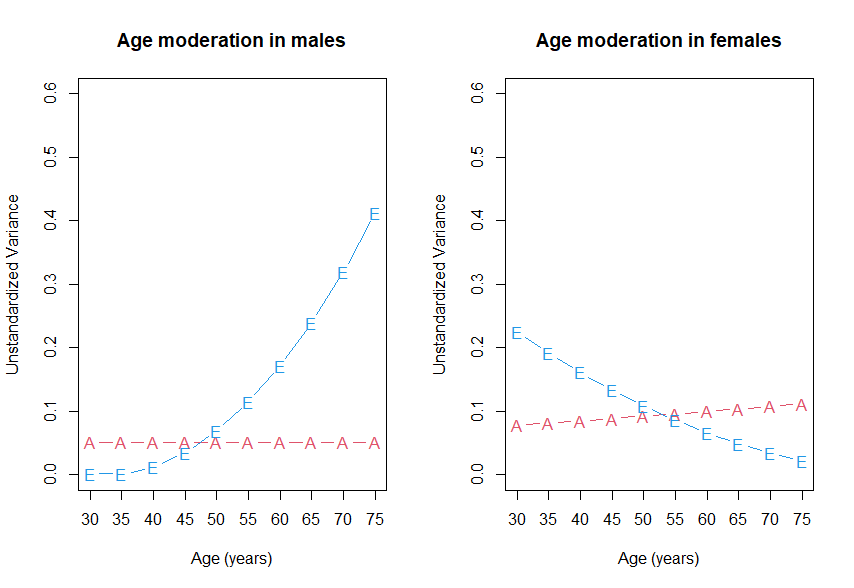


**B**

**A**

**eFigure 3:** Age moderation effect on the genetic and environmental contributions to plasma phosphorylated-tau 217 concentrations in males and females. The change in unstandardized variance is plotted. The dashed red line represents the unstandardized additive genetic variance over age. The dashed blue line represents the unstandardized unshared environment variance over age. Figure A = Age moderation effect in males. Figure B = Age moderation effect in females.


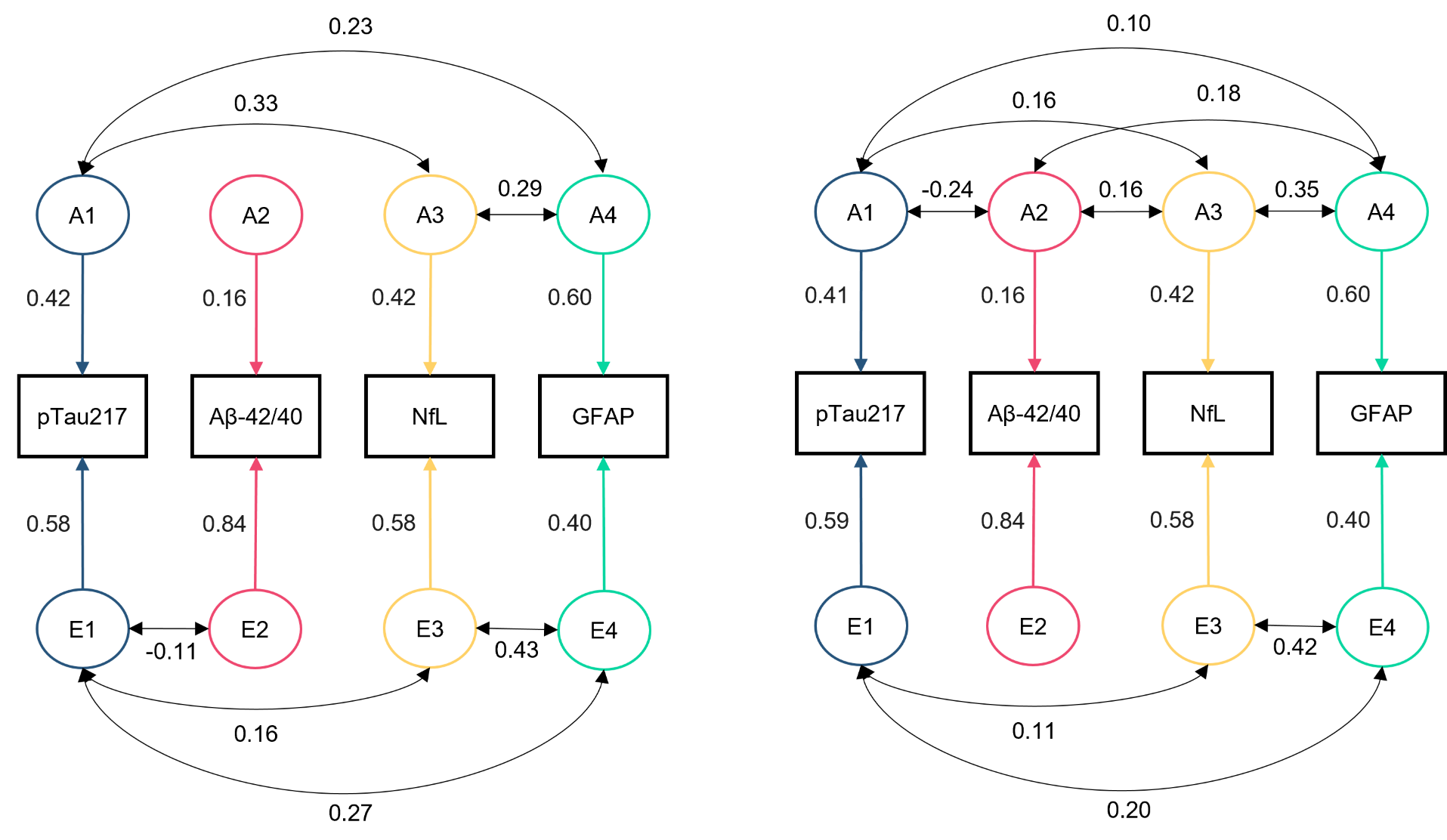


**B**

**A**

**eFigure 4. Path diagram of the multivariate extended design in males (A) and females (B).**

Single headed arrows represent paths. Double head arrows represent correlations.

| **eTable 3. Summary of the multivariate model in males.** | | | | | | | | | |
| --- | --- | --- | --- | --- | --- | --- | --- | --- | --- |
|  |  | **rA** | **rA%** | **rE** | **rE%** | **df** | **ΔLL** | **AIC** | **p-value** |
| **pTau217 +**  **NfL** | **AE + rA, rE** | 0.34 [0.14 ; 0.52] | 60% | 0.16 [0.04 ; 0.28] | 40% | 6176 |  | 14868 |  |
|  | **AE + rE** | - | - | 0.31 [0.24 ; 0.38] | 100% | 6177 | 9.44 | 14875 | 0.002 |
|  | **AE + rA** | 0.51 [0.39 ; 0.64] | 100% | - | - | 6177 | 6.29 | 14872 | 0.01 |
|  | **AE** | - | - | - | - | 6178 | 78.9 | 14943 | 7.5e-18 |
| **pTau217 +**  **GFAP** | **AE + rA, rE** | 0.23 [0.06 ; 0.39] | 44% | 0.27 [0.14 ; 0.39] | 56% | 6176 |  | 14868 |  |
|  | **AE + rE** | - | - | 0.39 [0.31 ; 0.46] | 100% | 6177 | 6.45 | 14872 | 0.01 |
|  | **AE + rA** | 0.47 [0.37 ; 0.58] | 100% | - | - | 6177 | 15.7 | 14881 | 7.4e-05 |
|  | **AE** | - | - | - | - | 6178 | 100 | 14964 | 1.7e-22 |
| **pTau217 +**  **Aβ-42/40** | **AE + rA, rE** | -0.01 [-0.32 ; 0.33] | 3% | -0.11 [-0.22 ; 0.007] | 97% | 6176 |  | 14868 |  |
|  | **AE + rE** | - | - | -0.11 [-0.18 ; -0.04] | 100% | 6177 | 0.004 | 14866 | 0.95 |
|  | **AE + rA** | -0.23 [-0.45 ; -0.05] | 100% | - | - | 6177 | 3.39 | 14869 | 0.07 |
|  | **AE** | - | - | - | - | 6178 | 9.23 | 14874 | 0.007 |
| **NfL + GFAP** | **AE + rA, rE** | 0.29 [0.13 ; 0.43] | 40% | 0.43 [0.31 ; 0.53] | 60% | 6176 |  | 14868 |  |
|  | **AE + rE** | - | - | 0.51 [0.43 ; 0.58] | 100% | 6177 | 6.50 | 14872 | 0.01 |
|  | **AE + rA** | 0.60 [0.51 ; 0.69] | 100% | - | - | 6177 | 40.3 | 14906 | 2.2e-10 |
|  | **AE** | - | - | - | - | 6178 | 76.9 | 14941 | 2.0e-17 |
| **NfL +**  **Aβ-42/40** | **AE + rA, rE** | -0.07 [-0.38 ; 0.23] | 100% | 0.03 [-0.09 ; 0.14] | 0% | 6176 |  | 14868 |  |
|  | **AE + rE** | - | - | 0.005 [-0.07 ; 0.08] | 100% | 6177 | 0.24 | 14866 | 0.62 |
|  | **AE + rA** | 0.01 [-0.17 ; 0.22] | 100% | - | - | 6177 | 0.61 | 14866 | 0.43 |
|  | **AE** | - | - | - | - | 6178 | 0.64 | 14864 | 0.73 |
| **GFAP +**  **Aβ-42/40** | **AE + rA, rE** | -0.11 [-0.39 ; 0.14] | 100% | 0.01 [-0.10 ; 0.13] | 0% | 6176 |  | 14868 |  |
|  | **AE + rE** | - | - | -0.02 [-0.10 ; 0.06] | 100% | 6177 | 0.65 | 14866 | 0.42 |
|  | **AE + rA** | -0.07 [-0.27 ; 0.11] | 100% | - | - | 6177 | 0.21 | 14866 | 0.65 |
|  | **AE** | - | - | - | - | 6178 | 0.70 | 14864 | 0.70 |

*A = Additive genetic effects. E = Environmental effects. rA = Correlation between genetic factors. rE = Correlation between environmental factors.. rA% = Proportion of the phenotypic correlation which is explained by the genetic correlation. rE % = Proportion of the phenotypic correlation which is explained by the environment correlation. df = degrees of freedom. ΔLL = Difference in log-likelihood.*

| **eTable 4. Summary of the multivariate model in females.** | | | | | | | | | | | | | | | | | |
| --- | --- | --- | --- | --- | --- | --- | --- | --- | --- | --- | --- | --- | --- | --- | --- | --- | --- |
|  |  | **rA** | | **rA%** | | **rE** | | **rE%** | | **df** | | **ΔLL** | | **AIC** | | **p-value** | |
| **pTau217 +**  **NfL** | **AE + rA, rE** | 0.16 [0.05 ; 0.27] | | 54% | | 0.11 [0.03 ; 0.19] | | 46% | | 12913 | |  | | 31990 | |  | |
|  | **AE + rE** | - | | - | | 0.19 [0.14 ; 0.24] | | 100% | | 12914 | | 7.70 | | 31996 | | 0.006 | |
|  | **AE + rA** | 0.27 [0.20 ; 0.35] | | 100% | | - | | - | | 12914 | | 8.00 | | 31996 | | 0.005 | |
|  | **AE** | - | | - | | - | | - | | 12915 | | 54.7 | | 32041 | | 1.3e-12 | |
| **pTau217 +**  **GFAP** | **AE + rA, rE** | 0.10 [0.001 ; 0.20] | | 34% | | 0.20 [0.12 ; 0.28] | | 66% | | 12913 | |  | | 31990 | |  | |
|  | **AE + rE** | - | | - | | 0.26 [0.20 ; 0.31] | | 100% | | 12914 | | 3.93 | | 31992 | | 0.047 | |
|  | **AE + rA** | 0.27 [0.20 ; 0.34] | | 100% | | - | | - | | 12914 | | 25.1 | | 32013 | | 5.5e-07 | |
|  | **AE** | - | | - | | - | | - | | 12915 | | 78.9 | | 32065 | | 7.4e-18 | |
| **pTau217 +**  **Aβ-42/40** | **AE + rA, rE** | -0.30 [-0.53 ; -0.10] | | 100% | | 0.03 [-0.04 ; 0.11] | | 0% | | 12913 | |  | | 31990 | |  | |
|  | **AE + rE** | - | | - | | -0.06 [-0.11 ; -0.01] | | 100% | | 12914 | | 9.02 | | 31997 | | 0.003 | |
|  | **AE + rA** | -0.24 [-0.38 ; -0.11] | | 100% | | - | | - | | 12914 | | 0.66 | | 31989 | | 0.42 | |
|  | **AE** | - | | - | | - | | - | | 12915 | | 14.5 | | 32001 | | 0.001 | |
| **NfL + GFAP** | **AE + rA, rE** | 0.35 [0.27 ; 0.43] | | 49% | | 0.42 [0.35 ; 0.48] | | 51% | | 12913 | |  | | 31990 | |  | |
|  | **AE + rE** | - | | - | | 0.56 [0.52 ; 0.60] | | 100% | | 12914 | | 48.8 | | 32037 | | 2.8e-12 | |
|  | **AE + rA** | 0.62 [0.56 ; 0.68] | | 100% | | - | | - | | 12914 | | 106 | | 32094 | | 8.5e-25 | |
|  | **AE** | - | | - | | - | | - | | 12915 | | 243 | | 32229 | | 1.7e-54 | |
| **NfL +**  **Aβ-42/40** | **AE + rA, rE** | 0.21 [0.03 ; 0.40] | | 100% | | -0.03 [-0.10 ; 0.05] | | 0% | | 12913 | |  | | 31990 | |  | |
|  | **AE + rE** | - | | - | | 0.06 [0.01 ; 0.10] | | 100% | | 12914 | | 7.81 | | 31996 | | 0.005 | |
|  | **AE + rA** | 0.16 [0.04 ; 0.28] | | 100% | | - | | - | | 12914 | | 0.57 | | 31989 | | 0.45 | |
|  | **AE** | - | | - | | - | | - | | 12915 | | 12.0 | | 31998 | | 0.002 | |
| **GFAP +**  **Aβ-42/40** | **AE + rA, rE** | 0.22 [0.07 ; 0.39] | | 100% | | -0.03 [-0.11 ; 0.05] | | 0% | | 12913 | |  | | 31990 | |  | |
|  | **AE + rE** | | - | | - | | 0.03 [-0.04 ; 0.09] | | 100% | | 12914 | | 4.79 | | 31993 | | 0.03 |
|  | **AE + rA** | | 0.18 [-0.07 ; 0.31] | | 100% | | - | | - | | 12914 | | 0.59 | | 31989 | | 0.44 |
|  | **AE** | | - | | - | | - | | - | | 12915 | | 6.06 | | 31992 | | 0.048 |

*A = Additive genetic effects. E = Environmental effects. rA = Correlation between genetic factors. rE = Correlation between environmental factors.. rA% = Proportion of the phenotypic correlation which is explained by the genetic correlation. rE % = Proportion of the phenotypic correlation which is explained by the environment correlation. df = degrees of freedom. ΔLL = Difference in log-likelihood.*

1. Xie X, Sun X, Wang Y, Lehner B, Li x. Dominance vs epistasis: the biophysical origins and plasticity of genetic interactions within and between alleles. nature communications. 2023;14:5551.

2. Neale MC, Cardon LR. Methodology for Genetic Studies of Twins and Families. Dordrecht, the Netherlands: Kluwer Academic Publishers; 1992.
